# Developing a process evaluation model in the GASP (groups for alcohol-misusing short-term prisoners) trial

**DOI:** 10.1186/1745-6215-16-S2-P9

**Published:** 2015-11-16

**Authors:** Yvonne Moriarty, Rachel McNamara, Michael Robling, Anna Kissell, Rebecca Playle, Pamela Taylor

**Affiliations:** 1 South East Wales Trials Unit, Cardiff University, Cardiff, UK; 2 Psychological Medicine, Cardiff University, Cardiff, UK

## 

Conducting a process evaluation of a complex intervention allows researchers to examine mechanisms of change not captured elsewhere. The MRC has published guidance[1] to support development of a streamlined approach and for reporting across public health interventions generally. Given the complex nature of the prison environment and in particular the many contextual factors that can impact on intervention delivery, a process evaluation is essential in a trial conducted in this setting. However, little guidance exists on the tailoring of process evaluations for complex environments where contextual factors may have a particularly significant impact. In this paper we present a process evaluation model for the Groups for Alcohol-misusing Short-term Prisoners (GASP) trial, following MRC guidance.

GASP is a two armed RCT of a group-based intervention (9 sessions over 3 weeks), drawing on motivational interviewing and skills development, to increase internal locus of control and reduce relapse among short-term alcohol misusing prisoners. Both qualitative and quantitative data are collected to help determine intervention mediators. Particular focus is placed on unique contextual factors which may impact significantly on reach, fidelity, exposure, recruitment, retention and contamination of the intervention (e.g. organisational factors such as availability of prison staff to transport prisoners to sessions and subsequent adherence). Theoretical assumptions underlying the intervention will also be examined, such as the degree to which any effect on the primary outcome (locus of control) is mediated by stages of change. This approach is novel in this setting and in trials of exceptionally vulnerable groups.
